# Oleanolic Acid Mitigates 6-Hydroxydopamine Neurotoxicity by Attenuating Intracellular ROS in PC12 Cells and Striatal Microglial Activation in Rat Brains

**DOI:** 10.3389/fphys.2019.01059

**Published:** 2019-08-21

**Authors:** Zama N. P. Msibi, Musa V. Mabandla

**Affiliations:** ^1^Department of Human Physiology, School of Laboratory Medicine and Medical Sciences, College of Health Sciences, University of KwaZulu-Natal, Durban, South Africa; ^2^Laboratory Medicine and Medical Sciences, Nelson R Mandela School of Medicine, College of Health Sciences, University of KwaZulu-Natal, Durban, South Africa

**Keywords:** oleanolic acid, Parkinson’s, 6-hydroxydopamine, microglia, reactive oxygen species

## Abstract

Oleanolic acid (OA), a biologically active pentacyclic triterpenoid compound, has been implicated in a number of clinical benefits including antioxidant, and anti-inflammatory properties. OA has been previously shown to ameliorate the toxic effects of 6-hydroxydopamine (6-OHDA), however, the mechanism by which this effect is exhibited is not clearly understood. In the present study, we investigated the role of OA in attenuation of microglial activation in 6-OHDA induced Parkinsonian rat model. We also explored the ability of OA to attenuate 6-OHDA-induced intracellular reactive oxygen species (ROS), and thus prevent cell death in PC12 cells. We accessed the utility of immunohistochemistry to assess striatal microglial activation, where shape descriptors such as area, perimeter, Feret’s diameter, aspect ratio and solidity were determined using the Fiji ImageJ software. Intracellular ROS and cell viability were assessed in PC12 cells using the OxiSelect^TM^ Intracellular ROS Assay Kit and MTT assay, respectively. We found that microglial activation was decreased in rats pre-treated with OA prior to 6-OHDA insult as well as in rats treated with OA 1 day post 6-OHDA exposure when compared to untreated rats, as determined by shape descriptors. This finding was in correlation with significantly improved motor symptoms and increased striatal dopamine in treated rats as compared to non-treated rats. Flow cytometry assessment of PC12 cells revealed a decreased amount of intracellular ROS in cells pre-treated with OA 6 h prior to 6-OHDA exposure and cells treated with OA 1 h post 6-OHDA exposure, suggesting that OA provides neuroprotection in PC12 cells by removing intracellular ROS, thereby reducing oxidative stress. Our finding suggest that OA exhibits its neuroprotective effect by attenuating striatal microglial activation, which results in neuroinflammation that is implicated in Parkinson’s disease pathology. Further studies detailing the mechanism by which OA interacts with microglia may be useful in understanding the role of OA in attenuating neuroinflammation.

## Introduction

Parkinson’s disease (PD) is a neurodegenerative disorder characterized by a substantial loss of dopaminergic neurons in the substantia nigra pars compacta (SNc), subsequently leading to a marked decrease of striatal dopamine (Hirsch et al., 1988), which manifests as motor and non-motor symptoms. This condition can be mimicked through a number of animal models, injection of 6-hydroxydopamine (6-OHDA) in the medial forebrain bundle in rodents is one of the most prominent models of parkinsonism (Rodriguez-Pallares et al., 2007). Although the human condition in its complexity has not been fully represented by one single model, animal models have provided an important tool in understanding neuropathological features and treatment strategies of PD (Blesa and Przedborski, 2014). In this study, we investigate the mechanism by which oleanolic acid (OA) exerts its neuroprotective activity both *in vivo* and *in vitro.* OA (3β-hydroxyolean-12-en-28-ioc acid) has been isolated from a number of plant species for medicinal use, which is especially common in plants belonging to the Oleaceae family, one of which being *Olea europaea* (Simonsen and Ross, 1957). This biologically active pentacyclic triterpenoid compound has been implicated in a number of clinical benefits such as the hepatoprotective effect (Lui, 1995; Wang et al., 2010). Other biological activities ascribed to OA include anti-inflammation (Li et al., 2005), anticancerous (Hsu et al., 1997), antioxidant (Senthil et al., 2007), anti-allergic, antimutagenic, and antiviral activities (Aparecida Resende et al., 2006) among others. Ndlovu et al. (2014) showed OA to have a preconditioning effect on a 6-OHDA induced parkinsonian cell culture model, suggesting a neuroprotective property in OA. The use of OA in Parkinsonian models is relatively new, and has shown promise in the treatment of PD, as amelioration of motor symptoms in a 6-OHDA-induced rat model has been observed (Mabandla et al., 2015); moreover, LID subsided with continued treatment with OA (Ndlovu et al., 2016).

## Materials and Methods

Prior to experimentation, ethical clearance (AREC/065/015D) was obtained from the Animal Research Ethics Committee (AREC) of the University of KwaZulu-Natal.

### Animals and Oleanolic Acid (OA) Treatment

Thirty two male Sprague Dawley rats (250 – 300 g) were obtained from the Biomedical Resource Unit of the University of KwaZulu. The rats were housed in an environmentally controlled facility and provided with food and water *ad libitum*. After a 7 day acclimatization period, the rats were randomly divided into 4 groups (*n* = 8): (1) 6-OHDA-lesioned control, (2) saline control, (3) OA treatment 7 days prior to 6-OHDA lesion, and (4) OA treatment 1 day post 6-OHDA lesion. OA (100 mg/kg; Sigma-Aldrich, South Africa) was orally administered every other day to prevent hypoglycemia (Mabandla et al., 2015) for both group 3 and 4.

### Stereotaxic Surgery

Ketamine (125 mg/kg) and Xylazine (5 mg/kg) were used to anesthetize rats prior to surgery. 6-OHDA (10 μg/4μL; Sigma-Aldrich, United States), dissolved in 0.2% ascorbic acid (Sigma-Aldrich, United States), and 0.9% saline was steroetaxically injected into the medial forebrain bundle at the rate of 0.5 μL/min. The following coordinates from lambda were used: anterior, 4.7 mm; lateral, 1.6 mm; and ventral, 8.4 mm below the skull (Paxinos and Watson, 1986). All procedures (except where mentioned) were carried out according to the 6-OHDA lesion protocol by Mabandla et al. (2015).

### Behavioral Tests

Forelimb function (step alternating), forelimb akinesia (step), Limb use asymmetry (cylinder), and total locomotor activity (open field) tests were done 14 days post-treatment to assess the neurotoxic consequence of 6-OHDA and neuroprotective effect of OA on behavior. All behaviors were evaluated in a designated behavioral assessment room, where rats were allowed 1-h acclimatization period prior to behavioral testing. An additional period of 2 h was allowed in-between different behavioral assessments.

#### Forelimb Function Test

Two weeks post-treatment, rats underwent a forelimb function test to ascertain their ability to alternate their forelimbs. All procedure were performed as described by Khaing et al. (2013). Briefly, the animals were held on a table top in a forelimb-only bearing stance, with both their forelimbs touching the table top. A shift in their center of gravity was introduced, after a 5 s delay, by moving the rat frontward, which forced them to take a step forward. This task was repeated four time for each rat with each run. The animals were classified as alternators if they displayed a 75% or higher alternating ability.

#### Forelimb Akinesia Test

Assessing step length measures the extent of step initiation impairment caused by 6-OHDA (Mabandla and Russell, 2010). Tests were done on a table top with a rough surface and ruler for step measurements. Each rat was held by its trunk above the table, restraining one forelimb so that its weight rests on the forelimb being tested. The rat was forced to move by gently thrusting it forward and the length of the step taken was recorded. Each step measure was done in triplicate and the average was calculated.

#### Limb-Use Asymmetry Test

The limb-use (cylinder) test was used to evaluate locomotor asymmetry (Tillerson et al., 2001; Mabandla and Russell, 2010). Each animal was placed on a transparent plexiglass cylinder and was allowed to explore for 5 min. The forelimb used initially to make contact with the cylinder wall, vertical exploration or the floor when landing was recorded. The final forelimb preference percentage score was calculated with the use of the following formula:

$$\left[\left(C\,o\,n\,t\,r\,a+\frac{1}{2}\,B\,o\,t\,h\right)÷\left(C\,o\,n\,t\,r\,a+I\,p\,s\,i+B\,o\,t\,h\right)\right]\times 100$$

Where “Contra” is the forelimb contralateral to the 6-OHDA-lesioned hemisphere; “Ipsi” is the forelimb ipsilateral to the 6-OHDA-lesioned hemisphere and “Both” denotes the concurrent use of both contralateral and ipsilateral forelimbs.

#### Total Locomotor Activity (Open Field Test)

Total locomotor activity was measured using the open field test (Walsh and Cummins, 1976). A 1 m × 1 m open field enclosure (50 cm high), with the floor clearly divided into 25 squares by masking tape was used. Animals were placed in the center square, one at a time, and allowed to explore the field for 5 min, during which a video camera was used to record the animal’s movement in the open field apparatus. After each assessment, 70% ethanol was used to clean the enclosure. Total locomotor activity was reported as the sum of total number of line crosses and rearing (Brown et al., 1999).

### Animal Sacrifice by Decapitation and Transcardial Perfusion

One day post behavioral assessments, 6 animals per group were randomly selected for decapitation using a guillotine, the remaining animals were sacrificed via transcardial perfusion. Whole brains were harvested and placed in 0.9% saline slush for a few minutes following decapitation, after which both the left and right striatum were removed, separately placed in 1.5 ml Eppendorf tubes, weighed, and snap frozen in liquid nitrogen. Trunk blood was collected in EDTA-coated vacutainer tubes and centrifuged at 3500 rpm for 7 min (IsoLab, Iso 9001). Plasma and cells were collected in Eppendorf tubes and snap frozen in liquid nitrogen for future use. Animals that were sacrificed by transcardial perfusion were weighed and deeply anesthetized by intraperitoneal injection of Ketamine and Xylazine (125/5 mg/kg) prior to perfusion using ice cold phosphate buffered saline (Sigma-Aldrich, South Africa) followed by 4% paraformaldehyde (MERCK, Germany). The brains were removed and post-fixed in 4% paraformaldehyde for 24 h, followed by cryopreservation in 30% sucrose solution for 48 h (Mabandla and Russell, 2010). After cryopreservation, the brains were coated with optimal cutting temperature (OCT) compound (Feliu et al., 2016) freezing medium (SMM Instruments, South Africa) and frozen in liquid nitrogen vapor prior to being stored at −80°C for immunohistochemical analysis.

### Immunohistochemistry

Whole brain samples from transcardial perfusion were retrieved from the −80°C biofreezer for immunohistochemistry analysis. With the aid of OCT, the frozen brains were mounted onto a Leica CM185 cryostat (SMM Instruments, South Africa) set at −20°C. Serial, 8 μm thick, slices were cut, mounting every 5th section on Leica X-tra^TM^ Adhesive Micro slides (SMM Instruments, South Africa). Antigen retrieval was performed following the EDTA, pH 6.0 protocol by Ghatak and Combs (2014). Briefly, mounted slides were placed inside a glass coplin jar with EDTA, pH 6.0, after which the sealed coplin jar was boiled for 10 min in a 500 mL beaker filled with water. Following antigen retrieval, Iba1 (Biocom Africa, South Africa) immunostaining was performed following standard manufacturer’s protocol (Abcam, 2016), where the slides were washed twice with TBS (Sigma-Aldrich, South Africa) in 0.025% Triton X-100 for 5 min per wash (All wash steps were performed with TBS in 0.025% Triton X-100, 5 min per wash). The samples were blocked for 2 h at room temperature (RT) in 10% serum prepared with 1% BSA, after which the slides were drained and incubated overnight in primary antibody (Iba1) diluted in TBS with 1% BSA (1:4000). Following two washes, slides were incubated in 0.3% H_2_O_2_ in TBS for 15 min at RT. Goat Anti-Rabbit IgG H&L (Biocom Africa, South Africa), diluted in TBS with 1% BSA (1:2000) was applied to the samples and slides were incubated at RT for 1 h. The samples were developed with chromogen at RT for 10 min and gently washed with running tap water, dehydrated, and mounted on the Leica DM500 light microscope for analysis. Striatal microglia images were captured and analyzed using the Fiji ImageJ software.

### Dopamine Elisa

Frozen striatal samples were retrieved from the −80°C biofreezer and thawed at RT. The Elabscience dopamine ELISA kit (ANATECH Instruments, South Africa) was used to measure dopamine concentration and all procedures were carried out according to manufacturer’s instructions. Briefly, all samples were minced and washed with ice-cold PBS to remove any excess blood. Samples were homogenized with a sonicator in PBS according to tissue weight [tissue weight (g): PBS (mL) volume = 1:9]. The homogenates were then centrifuged for 5 min at 5000 × *g* and supernatants were collected. Standard working solutions were prepared in the first two columns of the ELISA plate (96 well-plate), each dopamine concentration was added in duplicate. Samples were added in triplicate in subsequent wells and 50 μL biotinylated detection Ab working solution was added in all wells, the plate was covered and incubated for 45 min at 37°C. Following 3 wash steps using 350 μL of wash buffer, 100 μL of HRP conjugate working solution was added in each well, the plate was covered and incubated for 30 min at 37°C. The solution was aspirated from the wells followed by 5 subsequent wash steps. To each well, 90 μL of substrate reagent was added, the plate was sealed and incubated for 15 min at 37°C, followed by the addition of 50 μL of stop solution into each well. The optical density of each well was measured using the SPECTROstar*^NANO^* micro-plate reader (BMG LABTECH) set to 450 nm.

### Cell Culture

The rat adrenal pheochromocytoma (PC12) cell line obtained from the department of Biochemistry of the University of KwaZulu-Natal was used to assess the ability of OA to mitigate 6-OHDA-induced intracellular reactive oxygen species (ROS) and improve cell survival. Low passage number (P21) PC12 cells were grown and maintained in RPMI medium (Sigma-Aldrich, South Africa) supplemented with 10% fetal bovine serum, 5% horse serum and 2% penicillin-streptomycin (Sigma-Aldrich, South Africa) solution (penicillin 10.000 U/mL, streptomycin 10.000 μg/mL) according to Ndlovu et al. (2014). When more than 80% confluence was reached, cells were trypsinized and seeded in 96-well microtiter plates (Whitehead Scientific, South Africa) at the density of 50000 cells per well. The cells were allowed a 12-h attachment period in standard growth conditions. These cells were subsequently used for ROS detection and cell viability assays.

#### Intracellular Reactive Oxygen Species

The OxiSelect^TM^ Intracellular ROS Assay Kit (CELL BIOLABS, South Africa) was used to measure intracellular ROS, all experimental procedures were performed according to manufacturer’s instructions. Briefly, after the 12-h attachment period, media was removed from all wells and discarded; cells were then gently washed twice with Dulbecco’s phosphate buffered saline (DPBS; Sigma-Aldrich, South Africa). To each well, excluding unstained control, 100 μL of 1X DCFH-DA diluted in serum-free RPMI medium, was added followed by incubation at 37°C for 1 h. Unstained control wells received 100 μL medium. The media was discarded and each well was treated according to their respective experimental groups. Experimental groups: (1) OA treatment 6 h prior to 6-OHDA exposure; (2) OA treatment 1 h post 6-OHDA exposure; (3) OA treatment alone, (4) 6-OHDA exposure alone, (5) hydrogen peroxide positive control, and (6) unstained control (cells that never received DCFH-DA, OA, or 6-OHDA treatments).

*OA treatment prior to 6-OHDA exposure*: PC12 cells were treated with OA (5 μM) 6 h prior to 1 h 6-OHDA (150 μM) exposure. OA-containing medium was removed prior to 6-OHDA treatment and media was changed post 6-OHDA exposure. Cells were left in normal growth medium for 24 h before cell viability and ROS assessment. *OA treatment 1 h post 6-OHDA exposure*: PC12 cells were exposed to 150 μM 6-OHDA toxin for 1 h. The toxin was removed and cells were immediately treated with OA (5 μM) in normal medium and incubated for 24 h. *OA treatment alone*: PC12 cells were incubated in media-dissolved OA (5 μM) for 24 h. *6-OHDA/hydrogen peroxide exposure*: PC12 cells were exposed to 150 μM 6-OHDA or hydrogen peroxide (positive control) for 1 h. The toxins were removed, replaced with normal medium and the cells were incubated for 24 h. Quantification of fluorescence was done using flow cytometry and fluorescence images were obtained using a fluorescence microscope (Supplementary Data). For flow cytometry, cells were trypsinized and placed in flow cytometry tubes. A minimum of 10000 PC12 cell events at a medium flow rate was counted for the quantification of intracellular ROS. For all experiments, the mean fluorescent intensity (MFI) of ROS was recorded as the geometric mean obtained from the respective flow cytometric histogram using Kaluza Analysis Software, version 1.3 (Beckman Coulter, United States). Non-treated unstained PC12 cells were used as a control, where non-fluorescing cells were gated (population A) and the MFI of ungated cells (population B) was obtained. The same protocol was applied to all experiments to obtain respective MFIs.

#### Cell Viability Assay

Following the 12-h attachment period, media was removed and cells treated according to experimental groups: (1) OA treatment 6 h prior to 6-OHDA exposure; (2) OA treatment 1 h post 6-OHDA exposure; (3) OA treatment alone, (4) 6-OHDA exposure alone, and (5) untreated control. All experimental wells were treated as above (sec section “Intracellular Reactive Oxygen Species”) and experimental procedures were carried out according to the protocol by Ndlovu et al. (2014). Briefly, the dimethyl thiazolyl diphenyltetrazolium (MTT; Sigma-Aldrich, South Africa) assay was used for detection of cell viability. Following the 24-h incubation period, 20 μL of MTT was added to each well and the cells were incubated for 3.5 h in an incubator set at 37°C. the media was then removed and cells were incubated for 15 min with 150 μL of dimethyl sulfoxide (DMSO). Absorbance was determined using the SPECTROstar*^NANO^* microplate reader at 630 nm wavelength.

### Statistical Analysis

GraphPad Prism version 5 (GraphPad Software Inc., United States) statistical analysis software package was used to analyze data obtained from behavioral assessment, dopamine ELISA assay, IHC shape descriptors and cell viability assay. GraphPad Prism version 7 was used to analyze MFIs obtained from flow cytometric analysis of ROS. All data underwent Shapiro-Wilk normality test, and subsequently analyzed using one-way analysis of variance (ANOVA) followed by Bonferroni’s multiple comparison test. All data were expressed as the Mean ± SEM and *p* < 0.05 was considered significant. Graph asterisks: ^∗^*p* < 0.05, ^∗∗^*p* < 0.01, ^∗∗∗^*p* < 0.001 when compared with barred groups.

## Results

### Forelimb Function Test

**FIGURE 1:**
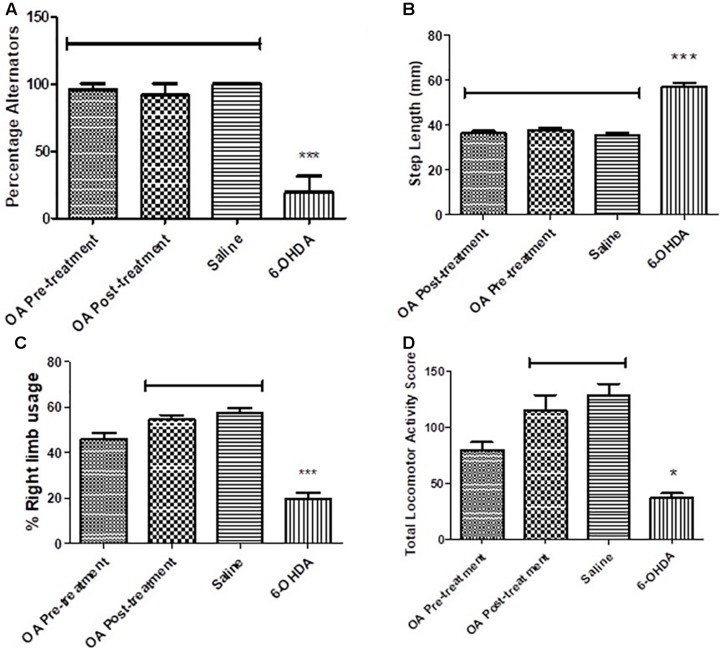
Behavioral tests in assessment of high dose 6-OHDA-induced neurobehavioral deficits in SD rats. **(A)** Forelimb function test showing a reduced ability to alternate forelimbs in untreated rats (*p* < 0.0001). **(B)** Forelimb akinesia test showing an increased average step length in untreated rats (*p* = 0.0008). **(C)** Limb-use asymmetry test showing a reduced preference use of the contralateral to lesion forelimb in untreated rats (*p* = 0.0005). **(D)** Open field test showing reduced total locomotor activity in untreated rats compared to the control (*p* = 0.0307).

Untreated 6-OHDA animals showed a significantly reduced ability to alternate their forelimbs while stepping forward, where two or more ipsilateral steps were taken in a row, compared to the control group and animals that received OA treatment (*p* < 0.0001, Figure 1A).

### Forelimb Akinesia Test

A 6-OHDA effect was observed in the average step length of the contralateral to lesion forelimb, OA treatment, and control groups had a shorter average step length when compared to the untreated 6-OHDA group (*p* = 0.0008, Figure 1B).

### Limb-Use Asymmetry Test

The cylinder test revealed a significant reduction in the use of the forelimb contralateral to 6-OHDA lesion as compared to the saline control and rats treated with OA 1 day post 6-OHDA lesion (*p* = 0.0005, Figure 1C). The ipsilateral forelimb was shown to be of preference to the 6-OHDA-lesioned rats (data not shown) in comparison to the saline control and the OA treated rats 1 day post 6-OHDA lesion.

### Total Locomotor Activity

An OA effect was observed in the total locomotor activity score of rats treated 1 day post lesion, in comparison to non-treated rats who displayed a markedly reduced locomotor activity (*p* = 0.0307, Figure 1D).

### Dopamine Concentration

**FIGURE 2:**
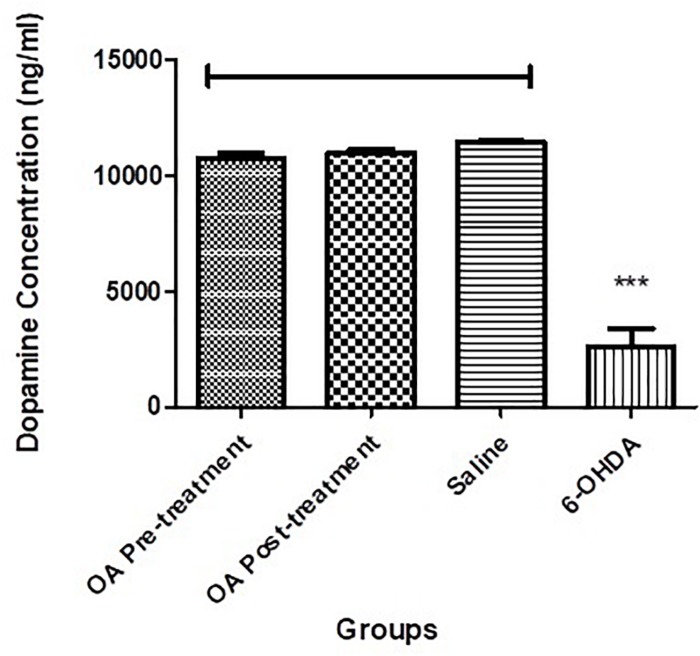
Dopamine ELISA assay showing significantly reduced striatal dopamine concentration in untreated 6-OHDA-lesioned SD rats (*p* < 0.0001), which was ameliorated by pre and post treatment with OA.

There was a significant reduction of dopamine in the left striatum of the 6-OHDA-lesioned rats, when compared to the compared to controls and OA treated groups (*p* < 0.0001, Figure 2).

### Immunohistochemistry

**FIGURE 3:**
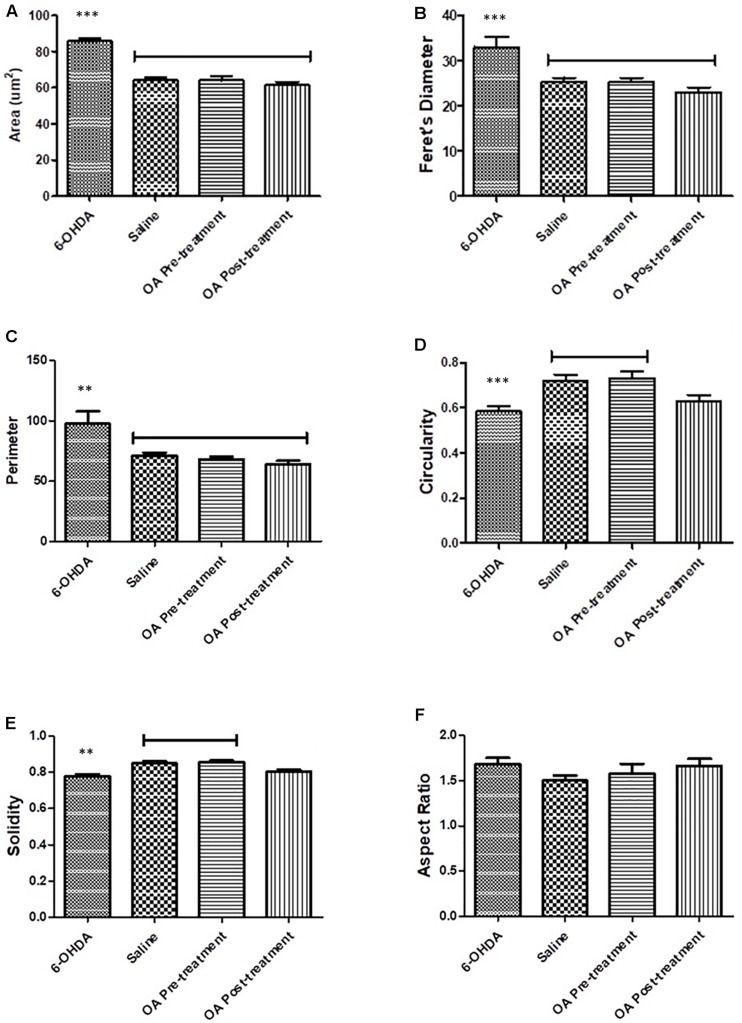
Shape descriptors analysis by the Fiji ImageJ software where six parameters were measure. **(A)** Area of microglia (*p* < 0.0001). **(B)** Feret’s diameter (*p* < 0.0001). **(C)** Perimeter (*p* = 0.0013). **(D)** Circularity (*p* = 0.0003). **(E)** Solidity (*p* < 0.0001). **(F)** Aspect ratio.

Morphological attributes of microglia assessed using Fiji imagej software were shape descriptors encompassing a number of parameters (Zanier et al., 2015). Striatal microglial area of untreated 6-OHDA-lesioned rats was significantly increased compares to saline and OA treated rats (*p* < 0.0001, Figure 3A). Other shape descriptors included Ferret’s diameter, which was found to be significantly increased (*p* < 0.0001, Figure 3B) in striatal microglia of untreated rats. Significant differences were also observed in perimeter (*p* = 0.0013, Figure 3C), circularity (*p* = 0.0003, Figure 3D) and solidity (*p* < 0.0001, Figure 3E). No significant differences were found in aspect ratio in all groups (Figure 3F).

### Intracellular Reactive Oxygen Species

**FIGURE 4:**
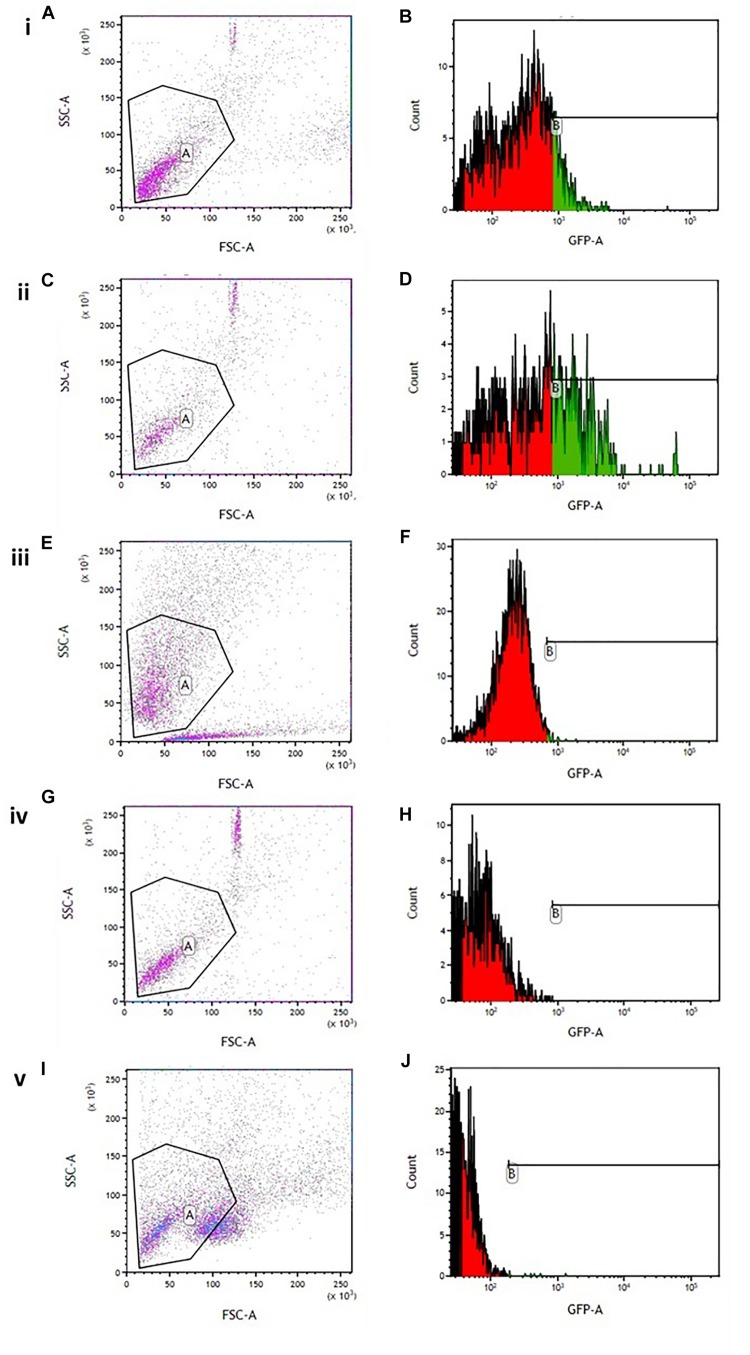
Flow cytometric analysis of intracellular ROS in 6-OHDA exposed PC12 cells. **(A**,**C**,**E**,**G**,**I)** Gating strategy used for all experiments. **(B)** 6-OHDA exposed cells (MFI = 1342). **(D)** Hydrogen peroxide treated cells (MF = 2191). **(F)** OA treated control (MFI = 403). **(H)** OA pre-treated cells 6 h prior to 6-OHDA exposure (MFI = 842). **(J)** OA treated cells 1 h post 6-OHDA exposure (MFI = 810).

**FIGURE 5:**
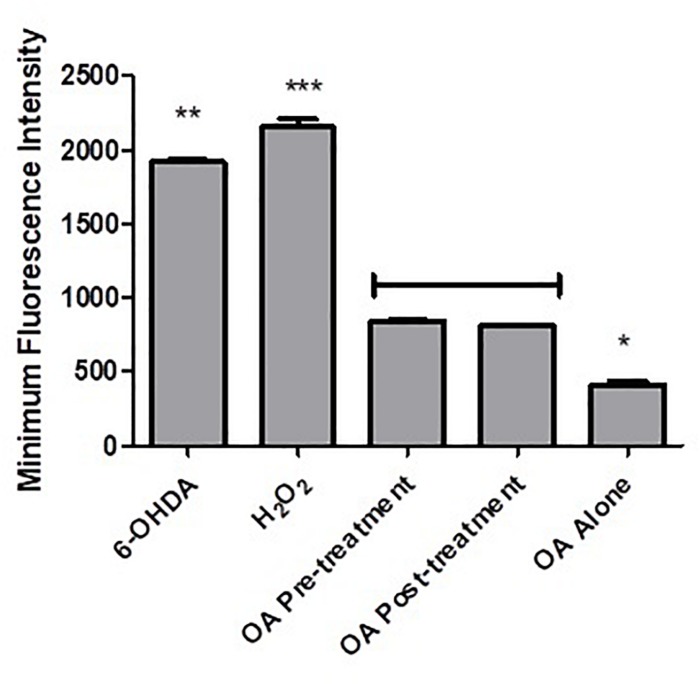
Comparison of MFIs obtained from flow cytometric analysis of intracellular ROS. A significant decrease in MFI was observed in both OA treated groups (*p* < 0.0001) when compared to 6-OHDA and H_2_O_2_ controls i, 6-OHDA; ii, H_2_O_2_; iii, OA alone; iv, OA pre-treatment; v, OA post-treatment.

Flow cytometry analysis showed an increased minimum fluorescence intensity (MFI) in 6-OHDA-treated PC12 cells (MFI = 1342, Figures 4A,B) and positive control cells treated with H_2_O_2_ (MFI = 2191, Figures 4C,D), compared to OA treated control cells (MFI = 403, Figures 4E,F), OA pre-treated cells 6 h prior to 6-OHDA exposure (MFI = 842, Figure 4H), and OA treated cells 1 h post 6-OHDA exposure (MFI = 810, Figures 4I,J). GraphPad Prism 7 statistical package was used to compare MFIs (Figure 5), and data were expressed as the Mean ± SEM and *p* < 0.05 was considered significant.

### Cell Viability

**FIGURE 6:**
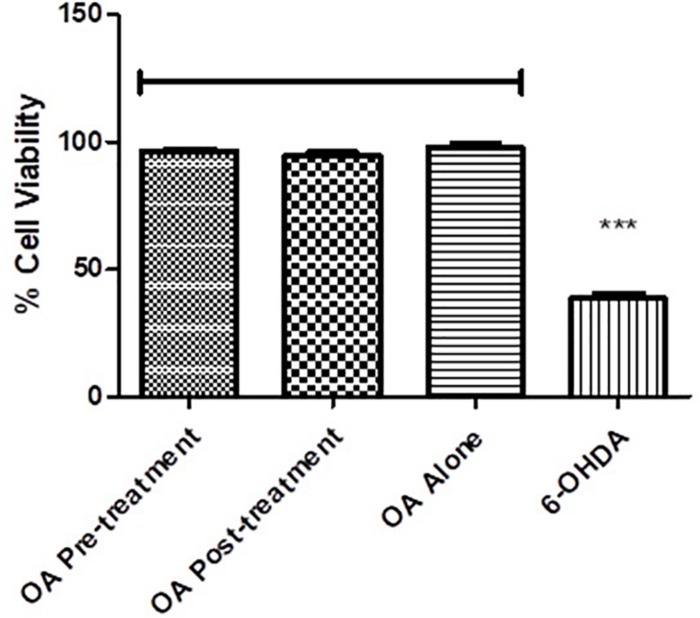
Cell viability assessment using the MTT assay. Cell viability is significantly reduced in 6-OHDA cells compared with OA control and OA treated pre and post 6-OHDA exposure cells (*p* = 0.0017).

PC12 cells exposed to 6-OHDA displayed less than 50% survival compared to the untreated control (*p* = 0.0017, Figure 6), while there were no significant differences between the untreated control and the OA treated cells (Figure 6).

## Discussion

We have previously shown OA, a pentacyclic triterpenoid compound, to ameliorate the neurodegenerative effects of the neurotoxin 6-OHDA (Mabandla et al., 2015), and demonstrated its ability to mitigate L-dopa induced dyskinesia (Ndlovu et al., 2016). However, the mechanism by which OA exhibit its neuroprotective effects is not clearly understood. Our current study sought to demonstrate some key areas of OA interaction, relative to PD pathogenesis. By means of four different behavioral tests, our study confirmed the neuroprotective effect of OA, by attenuating parkinsonian symptoms in Sprague Dawley rats (SD; Figure 1). We found that more than 50% of the untreated 6-OHDA-lesioned rats had a diminished ability to alternate their forelimbs, as shown through the forelimb function test (Figure 1A), which was ameliorated by pre and post-lesion treatment with OA. This is a relatively new test, originally shown to be a robust assessment of neurological deficits in cervical spinal cord injury (Khaing et al., 2013). The forelimb akinesia (step test), limb-use asymmetry (cylinder test) and open field tests (Figures 1A–D) have long been used in assessment of parkinsonism in rodents (Walsh and Cummins, 1976; Tillerson et al., 2001; Mabandla and Russell, 2010), and have been shown to be reliable measures of neurobehavioral deficits resulting from 6-OHDA neurotoxicity. This finding was further correlated by the significant increase in striatal dopamine concentration (Figure 2) in rats treated with OA pre and post exposure to 6-OHDA. We used a high dose of 6-OHDA (10 μg/4 μl) which resulted in more than 50% degeneration of striatal dopamine (Figure 2) which closely resembles the clinical condition of PD in humans (Bernheimer et al., 1973). 6-OHDA is thought to exhibits it’s neurotoxic effect via two mechanisms; (1) it’s highly oxidizable characteristic results in the subsequent accumulation of intracellular superoxide, hydroxyl radicals and hydrogen peroxide (Cohen and Heikkila, 1974; Blum et al., 2001; Blandini and Armentero, 2012), a ROS that causes oxidative damage to biomolecules (Halliwell, 2006; Jovanovic et al., 2016), following rapid auto-oxidation of the neurotoxin inside the cell (Blandini and Armentero, 2012); and (2) Direct inhibition of mitochondrial complex I and IV activity, as a result of accumulation of the neurotoxin (Glinka and Youdim, 1995; Blandini and Armentero, 2012). Wang et al. (2015) demonstrated the role of α-Synuclein in microglial migration regulation via hydrogen peroxide (H_2_O_2_), showing a preferential migration of mouse microglia toward H_2_O_2_, subsequently leading to microglial migration toward rat primary neurons. There is sufficient evidence implicating microglia in the pathogenesis of a number of neurodegenerative diseases (Hickman et al., 2018) including PD (Wu et al., 2002; Chung et al., 2010; Yan et al., 2014). Microglia are the principal innate immune cells in the central nervous system (CNS) and serve as the brain’s immune defense against infection and neuronal damage (Lull and Block, 2010). Under normal physiological conditions, microglia have been reported to play diverse roles in the brain including sensing and housekeeping (Hickman et al., 2018), as they form a complex traversing the CNS (Lawson et al., 1990). In their normal state, microglia are in their ramified morphology, scanning the environment for potential threat to the homeostatic brain environment; hence sensing is one of the essential roles of microglia as this is a requirement for their housekeeping functions, which include a neuroinflammatory response against pathogens, disease and injurious stimuli in the brain (Hickman et al., 2018). The exact OA-microglia interaction has not been defined, however, it has been demonstrated that microglia are activated prior neuronal damage resulting from injection of 6-OHDA into the medial forebrain bundle (Marinova-Mutafchieva et al., 2009). Our study suggests that OA may interact directly with microglia, attenuating microglial activation that would result in an unremitting neuroinflammatory response; and reduce oxidative stress by removing intracellular ROS (including H_2_O_2_), minimizing or preventing microglial migration toward neurons. α-Syn has been shown to bind to CD11b, a receptor expressed in microglia, via H_2_O_2_, resulting in directional migration of microglia to neurons (Wang et al., 2015), leading to subsequent neuronal damage. In an Alzheimer’s (AD) disease model, ursolic acid, a naturally occurring pentacyclic triterpenoid isomer of OA (Lui, 1995), has been shown to competitively bind to CD36 (Wilkinson et al., 2011), one of the receptors expressed in microglia (Huh et al., 1996), thereby preventing the binding of Aβ to CHO-CD36, a pathological feature of AD (Wilkinson et al., 2011). Our results also showed that OA inhibits the accumulation of ROS in PC12 cells (Figures 4, 5) and promote neuronal cell survival (Figure 6). As PC12 cells were independent from the physiological processes that generally occur in the CNS, our findings suggest that in addition to attenuation of microglial activation, which subsequently leads to accumulation of ROS, thereby creating oxidative stress seen in PD (Hwang, 2013), OA may exhibit its antioxidant activity by independently removing the injurious intracellular ROS in neuronal cells and promote neuronal cell survival. Our findings suggest that OA may exhibit its neuroprotective effects by attenuating microglial activation in high-dose 6-OHDA-lesioned SD rats, and independently remove intracellular ROS in PC12 cells, thereby promoting cell survival.

## Conclusion

In this study we have shown a possible interaction of OA and microglia in 6-OHDA-lesioned SD rats. We have also shown OA to independently remove intracellular ROS, thereby promoting neuronal cell survival. These finding were correlated by improved neurobehavioral deficits suggestive of parkinsonism in a rat model closely resembling the clinical PD condition. Our results further emphasize the potential of OA as an adjunct treatment for PD clinical conditions, and/or as a neurodegeneration preventive measure.

## Ethics Statement

Ethical approval (AREC/065/015D) was obtained from the Animal Research Ethics Committee (AREC) of the University of KwaZulu-Natal. All the experimental procedures were pre-approved by the Animal Research Ethics Committee (AREC) of the University of KwaZulu-Natal.

## Author Contributions

Both authors listed have made a substantial, direct and intellectual contribution to the work, and approved it for publication.

## Conflict of Interest Statement

The authors declare that the research was conducted in the absence of any commercial or financial relationships that could be construed as a potential conflict of interest.
