# Supplementary material for: Oleanolic Acid Mitigates 6-Hydroxydopamine Neurotoxicity by Attenuating Intracellular ROS in PC12 Cells and Striatal Microglial Activation in Rat Brains
Source: Front Physiol. 2019 Aug 21;10:1059. doi: 10.3389/fphys.2019.01059 (PMC6712087; doi:10.3389/fphys.2019.01059)
Supplement: Supplementary file 1 [file Table_1.DOCX]

Oleanolic acid mitigates 6-hydroxydopamine neurotoxicity by attenuating intracellular ROS in PC12 cells and striatal microglial activation in rat brains

Supplementary Data


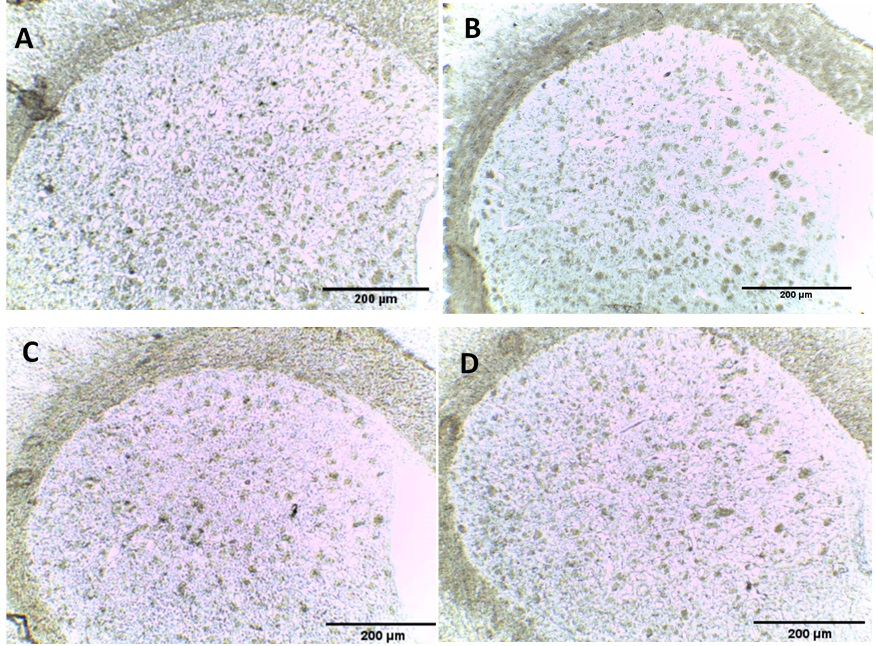


Figure 1: Light microscope images of the left striatum following immunochemistry processing. A) 6-OHDA; B) Saline control; C) OA treatment 7 days prior to 6-OHDA lesion; and D) OA treatment 1 day post 6-OHDA lesion.


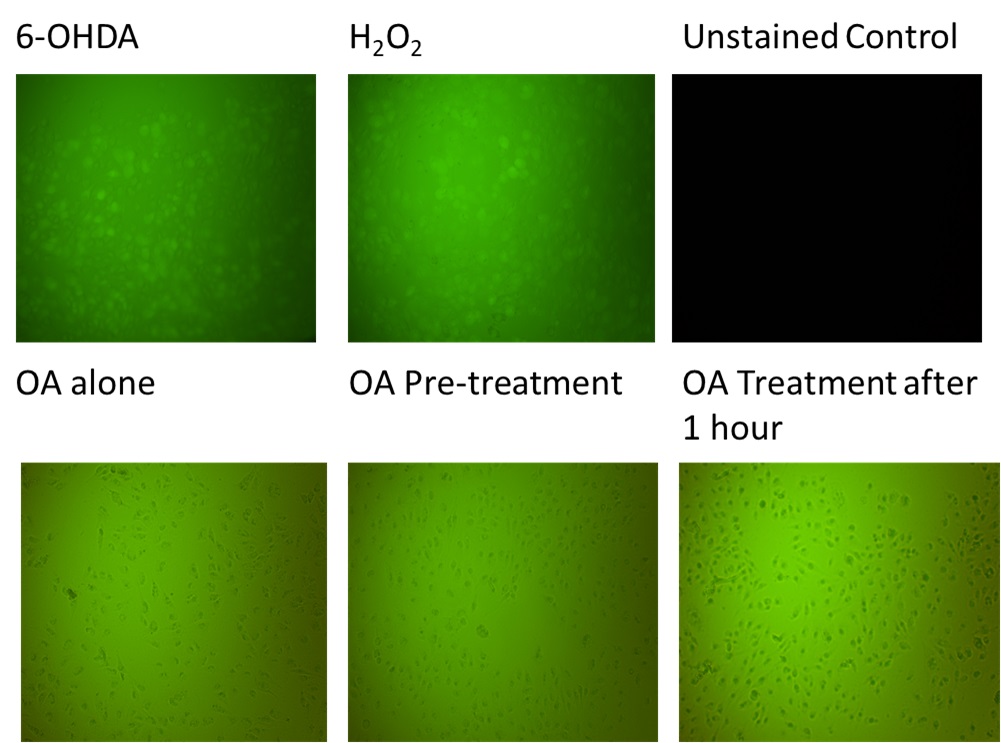


Figure 2: Fluorescence micrographs of PC12 cells stained with DCF-DA from the OxiSelect intracellular ROS assay kit, where fluorescence is observed from 6-OHDA and hydrogen peroxide treated cells.
